# Experimental Study on Bond Behavior of Glass Textile Mesh in Earth-Based Matrix

**DOI:** 10.3390/ma16031161

**Published:** 2023-01-29

**Authors:** Weinan Han, Feng Wu, Yugang Zhao, Haitao Wang, Shenglin Chu

**Affiliations:** 1 School of Civil Engineering, Dalian Jiaotong University, Dalian 116028, China; 2 Xinjiang Institute of Technology, Aksu 843000, China

**Keywords:** bond behavior, pull-out test, glass textile mesh, earth-based matrix, composites

## Abstract

The bond behavior between the textile and the earth-based matrix determined the reinforcement effectiveness of the composite systems. This paper presented a pull-out experimental study on the glass textile mesh reinforced earth-based matrix. The bond behavior was studied using different development length, mesh spacing size and matrix thickness, with a total of 32 experimental specimens. The test results showed that the peak pull-out force had increased by 31.7% and 40.5% with 200 mm and 300 mm versus 100 mm development length, respectively. The 16 mm compared to 10 mm matrix thickness specimens had a high strength improvement (9.73%) because the elevated thickness had increased the matrix strength. However, the 20 mm versus 10 mm mesh spacing size specimens had achieved a slight reduction (5.72%) due to the reduction in the number of textiles along the weft direction. The failure mode shifted from pulling out, compound modes (both pulling out and textile rupture) to textile rupture mainly accompanied by elevated development length. In addition, we discussed the applicability of the trilinear bond-slip model on the earth-based matrix and proposed a method based on the fracture energy concept for estimating the effective development length, which could provide a reference for future research.

## 1. Introduction

Earth is a cost-effective and locally available material in both conventional and monumental buildings, and earth buildings shelter almost one third of the world population or half the total population in developing countries. However, earth structures have the high seismic vulnerability as calamities caused by recent earthquakes demonstrated [1,2], and long-term environmental erosion triggers them to crack and collapse [3,4,5]. In the past, traditional strengthening techniques such as steel ties, buttresses or ring beams were largely used based on empirical knowledge, though the effect was not good [6]. Moreover, natural material meshes [7], geo-nets [8] and synthetic mesh [9] were also applied to the elements externally covered earthen matrix, which produced an improved mechanical effect. To improve erosion resistance of the structure, some researchers mixed inorganic or organic consolidation agents [10,11] into earthen materials.

In the last few years, reinforcement measures for structure repairs [12,13] have occurred in the literature. Fabric-reinforced cementitious matrix (FRCM), defined as a composite material consisting of cement-based matrix, such as mortar and concrete, and textile meshes had proven to be a promising composite for the strengthening of existing reinforced masonry or concrete structures [14,15,16]. Textile reinforced earth-based matrix (TRM) composite compatible with earth structures had emerged as an alternative strengthening solution because of its low self-weight, high tensile strength and ductility capacity, as already demonstrated for earth structures in many studies. Cassese, P., et al. [17,18] and Meybodian, H., et al. [7] found the reinforcement significantly improved the in-plane lateral behavior, energy dissipation capacity and found it had an average ductility capacity in adobe walls. Allahvirdizadeh, R., et al. [19] investigated the seismic in-plane behavior of reinforced adobe walls by means of advanced nonlinear finite element modelling. Sadeghi, H., et al. [20] found the reinforcement substantially improved the load-bearing capacity and ductility of adobe vaults under increasing vertical displacement. In addition, this reinforcement method had been applied to practical projects [21]. The above review showed the possibility to obtain a considerable reduction in mechanics vulnerability of adobe masonry structures by means of TRM systems made of earth-based matrix and textile, and also showed continuous textile reinforced earth-based mortar was a new class of material, which can be more suitable for earth structures. Furthermore, the local bond-slip relationship could be seen as a consequence of different failure mechanisms, such as debonding between the system and substrate or between the fabric and matrix.

Extensive research focused on the bond behavior of textile in cement-based mortar with diverse factors including the fabric type [22,23], quantity of yarn [24], weaving method [25], geometry and surface condition of the fabric [26], matrix type [27], and fabrication method [28]. Moreover, the bond-slip relationship was extracted, which led to an analytical [29,30] or a numerical [31,32] simulation that fitted well with the experimental results. However, limited research was available concerning the bond behavior of textiles in earth-based mortar. Romanazzi, A., et al. [33] investigated different types of meshes (glass and nylon), bonded lengths and testing speed by pull-out test, and proposed an adhesion-friction law [34]. Rathod, B., et al. [35] focused on cement stabilized earth-based matrix with dry density, cement content, and moisture content and coir fibers with different embedment lengths by pull-out test. In addition, some research reported that bond behavior of other pull-out objects such as rebar [36], steel [37], bamboo [38] and timber [39] in cement stabilized earth-based matrix mainly with diverse factors including dry densities and content of the matrix as well as geometric properties and types of pull-out objects. Hence, current studies have not sufficient explored the earth-based matrix. Especially, there was limited research on the geometric properties of the matrix and textile, and the other materials stabilized the earth-based matrix.

**Figure 1:**
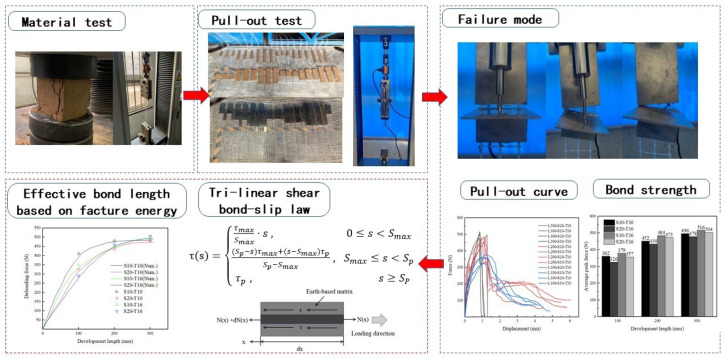
Flowchart.

In this case, this paper used the polyurethane chemical treatment stabilized earth-based mortar as a matrix material. This could help TRM to protect substrates from long-term environmental erosion and preserve the original appearance of historical buildings [40,41]. The glass textile mesh was used due to its high compatibility with low strength earth-based matrix [42] and low cost. Considering three development lengths, two mesh spacing sizes and two matrix thicknesses, a total of 32 specimens were carried on the pull-out test with an analysis of the failure mode, bond strength and the force versus displacement curves. Moreover, this paper discussed the applicability of the trilinear bond-slip model on the earth-based matrix and proposed a method based on the fracture energy theory for estimating the effective development length. Figure 1 presented the flowchart of this paper.

## 2. Experimental Program

### 2.1. Materials Properties

**Figure 2:**
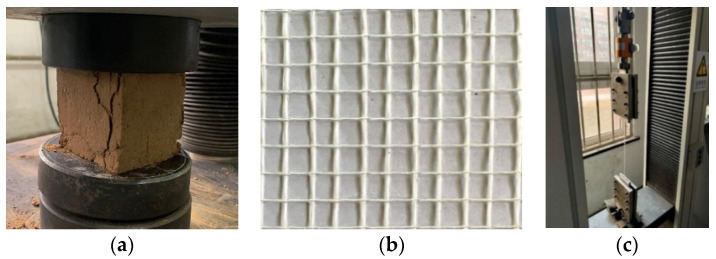
Preparation of the materials: (**a**) compressive test of earth-based mortar, (**b**) textile detail, (**c**) tensile test of single yarn.

The earth-based matrix was the mixture of the local representative soil, fine sand with a weight ratio of 1:1 (soil: sand), water with a weight ratio of 1:5 (water: soil plus sand), and 35% polyurethane [40,41] with a volume ratio of 1:4 (polyurethane: water) [43]. Through mechanical compaction, the relative movement of the soil and sand particles promoted the discharge or compression of air and water, thus reducing the pore volume and forming the occlusion and friction between the particles. Moreover, when the polyurethane stabilizer was diluted with water and then mixed with soil and sand, the polyurethane reacted rapidly with water to form a polymer, which filled the pores and wrapped the particles, and reacted with minerals to form the cementing force. The materials were bonded to form the whole structure by the above physical or chemical connection [44]. The mechanical properties of the earth-based matrix were characterized according to the requirement [45] by testing standard cubic specimens as shown in Figure 2a. The textile woven by unimpregnated glass fibers [46] as shown in Figure 2b were used as the reinforcement because glass textile mesh had low costs and strength compatible with earth-based matrix [42]. The mechanical properties of glass textile mesh were characterized by tensile tests according to the requirement [47] as shown in Figure 2c. Table 1 presented the mechanical properties of the earth-based matrix and glass textile mesh.

### 2.2. Specimens

**Figure 3:**
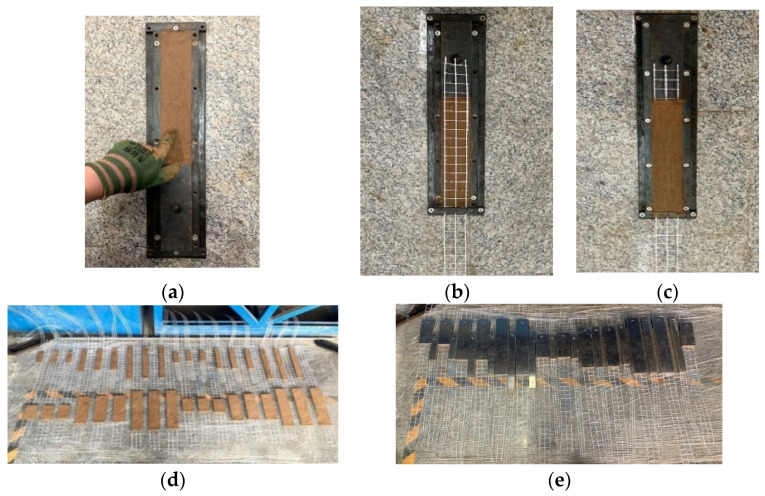
Samples preparation: (**a**) place a layer of the mortar, (**b**) place one layer of glass textile mesh, (**c**) place one layer of glass textile mesh, (**d**) demold curing, and (**e**) fix the aluminum plate.

The mesh spacing size, the matrix thickness and the development length of the glass textile mesh in the earth-based matrix were considered as research factors; respectively, 10 mm and 20 mm mesh spacing size, 10 mm and 16 mm matrix thickness, 100 mm, 200 mm and 300 mm development length. The mold and textile were prepared according to the test requirements. The standard plates were manufactured according to the following steps: (1) placed one layer of the mortar in a mold; (2) placed one layer of glass textile mesh on the mortar layer; (3) casted an additional layer of the mortar; (4) spread and compacted the matrix immediately after the vibration process. After one day, the plate was demolded and the specimens were cured under standard conditions for 28 days. Finally, on each specimen, the aluminum plate was fixed by glue with good adhesion and no invasion. This test setup was used to avoid the relatively thin earth-based matrix laterally splitting into several small segments during the pull-out process [33] and any difficulty in obtaining the expected development length. If the earth-based matrix incurred a fracture in the middle section, the development length would become the half test design. The specific production process of the specimen was shown in Figure 3. It is worth noting that the 10 mm mesh spacing size embedded in 30 mm wide matrix while 20 mm mesh spacing size embedded in 60 mm wide matrix, to ensure that remained three yarns in warp directions to change the density of the textile mesh in the mortar. This was supported by increasing mesh spacing size with increasing substrates area in the actual engineering.

### 2.3. Test Set-Up

**Figure 4:**
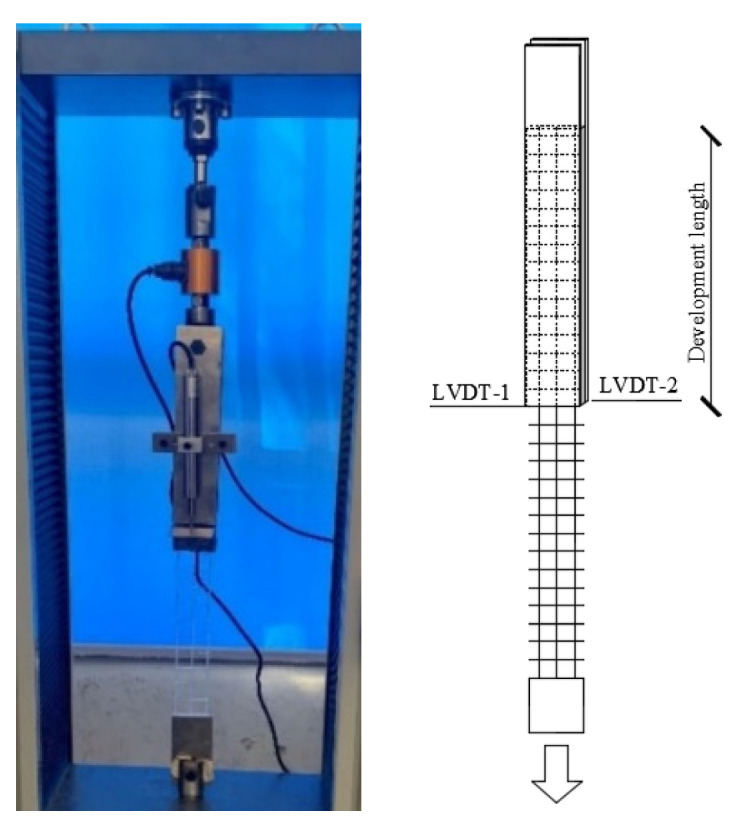
Pull-out test and configuration.

The specimens were divided into twelve series with three identical specimens for each series, namely “development length-textile spacing-matrix thickness-1, 2 or 3”. The detailed information was presented in Table 2. All specimens were cast with each end fitting clamps of testing machine. It was noted that specimens should be kept vertically to avoid any damage due to bending. Finally, the pull-out tests were carried on an electronic universal test machine at a constant displacement rate of 1.0 mm/min. Two linear variable differential transformers (LVDTs) were retained by a holder, which consisted of two customized clamps fixed on the specimens, to measure the displacement of two L-shaped aluminum plate pasted on textile at loading end. The test setup is shown in Figure 4.

## 3. Experimental Results and Discussion

**Figure 5:**
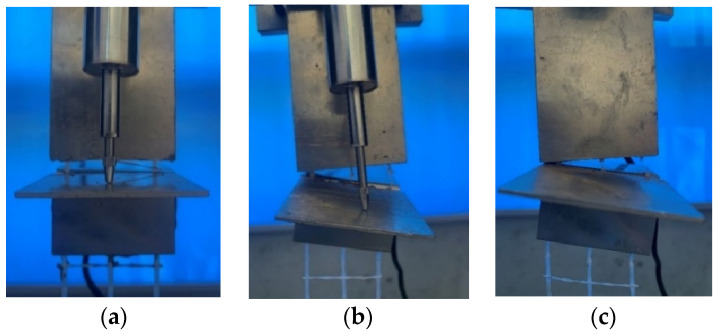
Failure mode: (**a**) pulling out, (**b**) compound mode, and (**c**) textile rupture.

**Figure 6:**
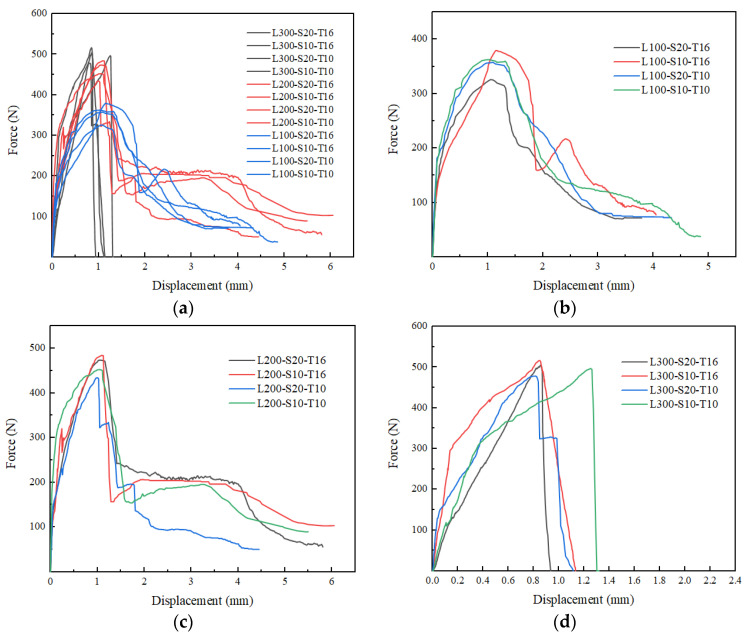
Force versus displacement curves: (**a**) all series, (**b**) 100 mm, (**c**) 200 mm, and (**d**) 300 mm development length.

First, this section analyzed the force versus displacement, the failure mechanism of the whole loading process and the final failure mode. Three failure modes had been observed during the tests illustrated in Figure 5. Figure 6 showed the average force versus displacement curves for three samples of all twelve series. Initially, the load was transmitted from the yarns to the matrix by adhesion; the curves showed a linear increasing trend. With displacement increasing, micro-cracks appeared in the matrix or matrix-textile interface and gradually developed along the matrix-textile interface resulting in debonding and friction was in action for the resistant mechanism in the debonding area; the pull-out stiffness decreased and curves showed a nonlinear relationship. With debonding length increasing, the adhesion gradually disappeared and friction increased in dynamic balance with load; the curves exhibited a softening decreasing trend shown in Figure 6b. These specimens exhibited relative slippage without observed filament or textile rupture (pulling out) in Figure 5a. However, when the load exceeded tensile strength of filament or textile in pulling out process, the curves showed a steep downward step in Figure 6c; these specimens presented compound mode in shown in Figure 5b. This phenomenon was also reported in the literature [22,24]. It was observed that a slight increase in load-bearing capacity such as L200-S10-T16, L200-S10-T10. This might be attributed to the aggregate interlocking and friction after the initiation of debonding, which was accompanied by large deformation. It is worth noting that the average curves included the curve of pulling out and compound mode specimens. The curve of L100-S10-T16 contained two compound mode specimens and the curve of L200-S20-T10 contained two pulling out specimens, hence they were different from the average curve of the same development length. In addition, when the adhesion mainly accounted for the resistant mechanism, the load already exceeded tensile strength of the textile; the curves exhibited steep drop as shown in Figure 6d; these specimens presented textile rupture as Figure 5c.

**Figure 7:**
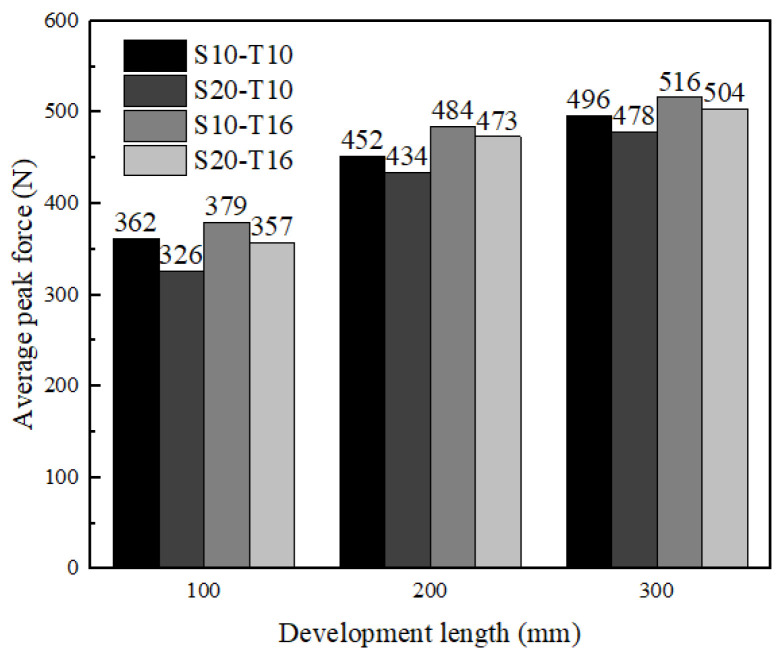
Average peak force of each series.

In addition, this section analyzed the difference of three research factors on the test results. The elevated development length could change the failure mode. As shown in Figure 5, the failure mode shifted from the pulling out, compound mode to textile rupture with the increase of development length. The specimens with 100 mm and 200 mm development length all exhibited pulling out and compound mode. However, seven specimens (L100-S10-T10-1/2/3, L100-S20-T10-1/2/3, L100-S20-T16-1) were pulled out with 100 mm development length while three specimens (L200-S20-T10-2, L200-S20-T10-3, L200-S10-T10-2) were only pulled out with 200 mm development length. All of the specimens with 300 mm development length exhibited textile rupture. It is clearly signified in Figure 7 that the load-carrying capacities were increasing continuously for all samples with increasing development length. The specimens with development length elevated from 100 mm to 200 mm had the obvious improvement (31.7%) in the peak load, while the specimens with development length elevated from 200 mm to 300 mm had a slight variation (8.3%). This presented 200 mm and 300 mm development length nearly reached the effective bond length of the composites. Additionally, it was interesting to note that textile rupture with 100 mm and 200 mm development length achieved the peak force lower than the strength capacity of the yarn as showed in Figure 7. This premature failure was attributed to successive breaking down of yarns from the sleeve to the core which prevented the full exploitation of the textile mesh capacity in the slippage progress [48]. This phenomenon was also mentioned in many studies [33,34,49]. The specimens with 300 mm development length had achieved textile mesh capacity due to less slippage. Figure 6a represented the force versus displacement behavior of tested samples from elevated (100 mm) to extreme (300 mm) development length. The general observations about the force versus displacement curves highlighted that the load-carrying capacities and stiffness characteristics were highly influenced by the elevated development length. This was consistent with other studies on earth-based matrix [33] and cement-based matrix [50,51].

The elevated mesh spacing size decreased the average peak force by 5.72% and the elevated thickness increased the value by 9.73% as shown in Figure 7. This was because the elevated mesh spacing size was accompanied by a reduction in the number of the textile along the weft direction [50] while the elevated thickness could increase the strength of the matrix to some extent [51]. Figure 6b–d compared the force versus displacement curve of tested samples at varying mesh spacing size and matrix thickness at the same development length. The comparative analysis proved that curves tended to soften in the debonding stage with increasing mesh spacing size. This was also supported by the failure mode in Table 2, among which the number of specimens, occurring textile rupture in the pull-out process (compound mode), with elevated mesh spacing size decreased from eight to six, while the number of specimens with elevated thickness increasing from five to nine. This showed that the mesh spacing size and matrix thickness also effected the failure mode to a certain extent.

## 4. Prediction Model

Taking into account the results of the experimental program above-mentioned, this section further analyzed the corresponding analytical bond-slip law. The existing research on the relatively accurate bond-slip model of fiber and cement-based mortar included tri-linear shear bond-slip law [29,52], four-linear shear bond-slip law proposed by Shilang, Xu, et al. [53] and elastic-brittle bond-slip law proposed by Pierluigi, et al. [54]. Tri-linear shear bond-slip law had wide applications, had a more simple and brief formula compared with four-linear shear bond-slip law and could reflect plastic characteristics of earth-based matrix compared with elastic-brittle bond-slip law. Hence, this paper studied the applicability of the tri-linear shear bond-slip law on earth-based matrix. The tri-linear shear bond-slip law included three stages as shown in Equation (1), namely the elastic stage in which the shear stress linearly increased with slip, the debonding stage in which the shear stress decreased linearly after reaching the maximum shear stress $\tau_{max}$ and the friction stage in which the shear stress remained constant $\tau_{p\;}$.

(1) $$\tau \left(s\right)=\left\{\begin{array}{cc} \frac{\tau_{max}}{S_{max}}\cdot s, & 0\leq s<S_{max}\\ \frac{\left(S_{p}-s\right)\tau_{max}+\left(s-S_{max}\right)\tau_{p}}{S_{p}-S_{max}}, & S_{max}\leq s<S_{p}\\ \tau_{p}, & s\geq S_{P} \end{array}\right.$$

where s was the relative slip of the textile and matrix, and τ(s) was the local shear stress on the textile-matrix interface. $S_{max}$ and $S_{p}$ were the slip values corresponding to $\tau_{max}$ and $\tau_{p\;}$.

**Figure 8:**
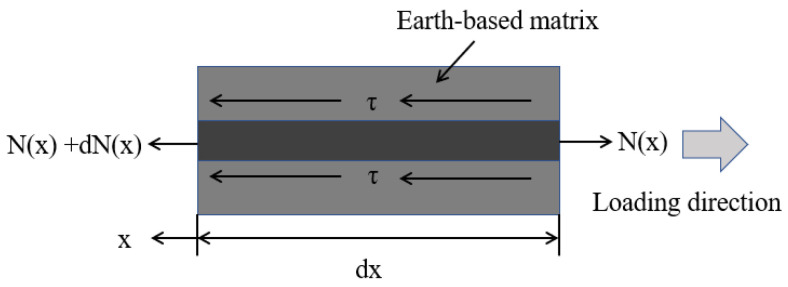
Static scheme of the interface during the pull-out test.

First, considering the static equilibrium along the development length and the infinitesimal interface length dx, the equilibriums could be expressed as Equation (2) and Equation (3). Then, substituting the Equation (3) into Equation (2) lead to Equation (4). Finally, substituting Equation (1), Equation (4) and the boundary conditions of each stage into Equation (3) obtained the force versus displacement relationships. Figure 8 shown static scheme of the interface during the pull-out test. Table 3 showed characteristic values in the bond-slip law.

(2) $$E_{f}A_{f}ds/dx=dN\left(x\right)$$

(3) dN(x)=cτ(s)dx

(4) $$d^{2}s/dx^{2}-c\tau \left(s\right)/E_{f}A_{f}=0$$

where 𝐹(x) was tensile force, c was the perimeter of the yarn [55], 𝜏(𝑥) was shear stress at textile-matrix interface in the section x, $E_{f}$ and $A_{f}$ were the elasticity modulus and cross-sectional area of a single yarn.

**Figure 9:**
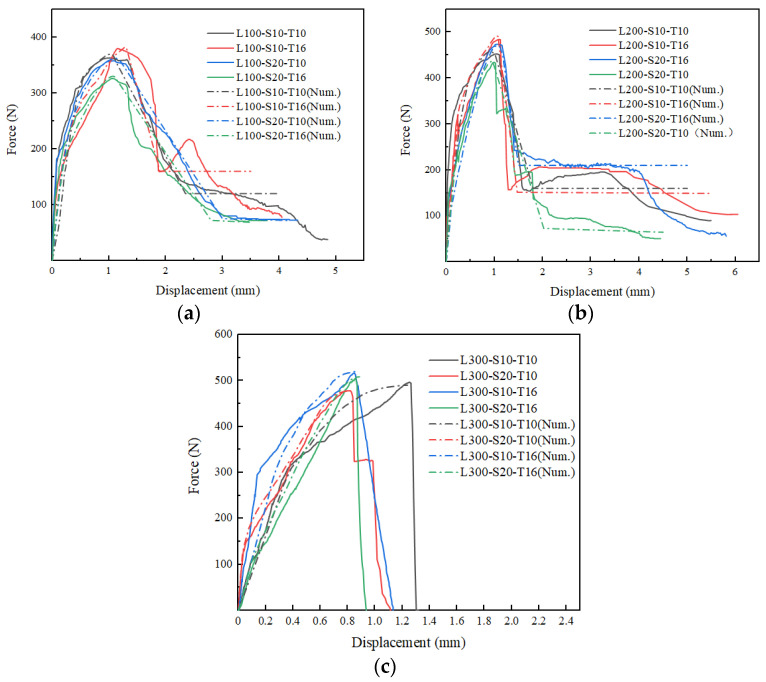
Theoretical curves and experimental curves with the varying development length: (**a**) 100 mm, (**b**) 200 mm, (**c**) 300 mm.

Figure 9 compared the average experimental curves with theoretical curves. The comparison results showed the theoretical curves had good agreement with experimental curves in ascending section. In particular, the theoretical curves simulated the characteristic of load softening trend and precisely predicted the peak force. However, there was some deviation in the descending section. The linearization of the theoretical curves could not precisely reflect the gradually softening load trend in the pulling out characteristic curves such as L100-S10-T10, L100-S20-T10, L100-S20-T16 and L200-S20-T10. The theoretical curves could not simulate steep downward step shape in the compound mode characteristic curves such as L100-S10-T16, L200-S10-T16, L200-S10-T10 and L200-S20-T16. Furthermore, the theoretical curves in Figure 9c had no descending section because 300 mm development length specimens had no friction stage. To sum up, the tri-linear shear bond-slip law could reflect overall trend of the bond behavior of glass textile mesh in earth-based matrix, providing reliable reference for further theoretical analysis on the study.

In addition, there was an effective bond length $L_{eff}$ of glass textile mesh in earth-based matrix, beyond which the bonding strength could not increase with the development length increase, as reinforcements externally bonded to concrete [56,57]. First, calculating Equation (1) and Equation (4) obtained shear stress τ(s) when x = L. Then, substituting τ(s) into Equation (5) obtained the interfacial fracture energy $G_{f}$. Finally, substituting $G_{f}$ into Equation (6) obtained the debonding force $P_{dbu}$ corresponding to the development length L.

(5) $$G_{f}=\int_{0}^{\infty }\tau \left(s\right)ds$$

(6) $$P_{dbu}=\sqrt{2cE_{f}A_{f}\cdot G_{f}}$$

**Figure 10:**
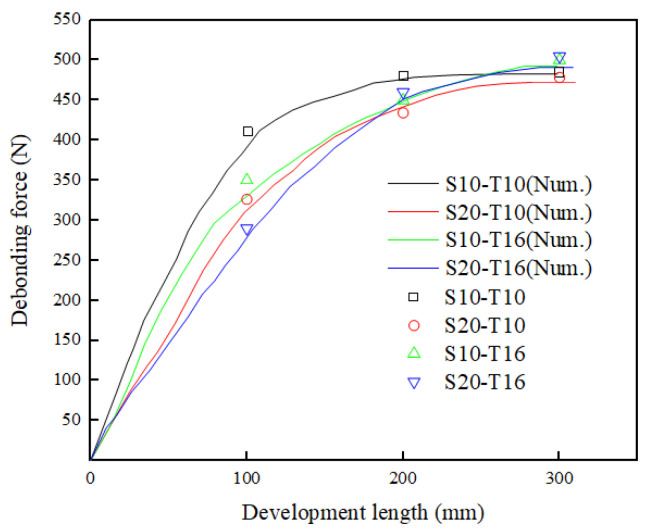
Comparison between experimental data and theoretical debonding force versus development length curves.

Figure 10 compared the theoretical debonding force versus development length curves with experimental data. Comparisons indicated the curves could accurately predicted experimental data. It could be observed that the debonding force was increasing with increasing development length but the increasing rate gradually decreased. This phenomenon was consistent with literature [58]. When development length approached 300 mm, the curves gradually tended to a continuous softening curve. Combined with previous analysis on the failure mode, 300 mm development length could be effective bond length of glass textile mesh in the earth-based matrix. At this time, the tensile properties of the glass textile mesh gave full play to its effectiveness.

## 5. Conclusions

This work investigated the bond behavior of glass textile mesh in earth-based matrix with varying development length, spacing mesh size and matrix thickness, analyzed different parameters, such as failure modes, load-carrying capacities and the force versus displacement curves, and discussed the tri-linear shear bond-slip law on earth-based matrix and proposed the effective bond length based on fracture energy concept. The summary remarks were listed as following:

(1) The research found three different failure modes, respectively, pulling out, textile rupture and compound mode with pulling out and textile rupture. The development length was main factors affecting the different failure mode. The specimens with 100 mm and 200 mm development length all appeared pulling out and compound mode, but more specimens with 200 mm occurred compound mode. Especially, when the development length reached 300 mm, all of specimens exhibited textile rupture. In addition, the mesh spacing size and matrix thickness also had affected on failure mode to a certain extent.
(2) The research factors affected the load-carrying capacities to varying degrees. The 200 mm and 300 mm development length specimens had an obvious improvement in the average peak force by 31.7% and 40.5% compared with 100 mm development length. The 20 mm mesh spacing size had the reduction in the average peak force by 5.72% compared to 10 mm mesh spacing size due to the reduction in the number of the textile along the weft direction while the value had an increase by 9.73% from 10 mm to 16 mm the matrix thickness due to the increase of the matrix strength.
(3) The force versus displacement curves in the ascending section converted from the original linear to a nonlinear relationship due to micro-cracks gradually developing along the matrix/textile interface. In the descending section, the curves exhibited showed three different shapes according to different failure mode: a continuous softening trend (pulling out), a steep downward step (compound mode) and steep drop (textile rupture).
(4) Theoretical curves based on the tri-linear shear bond-slip law simulated the characteristic of load softening in ascending section and precisely predicted the peak force, even though there was some deviation in descending section, proving the feasibility of the law applied to earth-based matrix. Moreover, the proposed theory based on fracture energy concept estimated effective bond length of glass textile mesh in the earth-based matrix was 300 mm at least.

In summary, this study covered the configuration in a majority of manufactured products, and would obtain more general conclusion by considering more factors and numerical simulation in the future.

## Figures and Tables

**Table 1:** Mechanical properties of earth-based matrix and glass textile mesh [‘‘()” indicates COV].

Materials	Area of Warp Yarn (mm^2^)	Surface Mass Density (g/m^2^)	Strength (MPa)	Ultimate Strain (%)	Elastic Modulus (MPa)
Earth-based matrix	-	-	5.6 (3.35%)	3.07 (6.31%)	132.5 (6.74%)
Glass textile mesh	0.38	159	501.43 (5.12%)	3.62 (6.43%)	13,862 (3.53%)

**Table 2:** Summary of the results for all the test specimens.

ID	Failure Mode	Peak Force(N)	Strain at Peak Force (%)
L100-S10-T10-1	Pulling out	342.986	1.245
L100-S10-T10-2	Pulling out	368.940	0.974
L100-S10-T10-3	Compound mode	376.321	0.865
L200-S10-T10-1	Compound mode	463.547	1.137
L200-S10-T10-2	Pulling out	441.136	0.988
L200-S10-T10-3	Compound mode	452.628	1.043
L300-S10-T10-1	Textile rupture	487.365	1.188
L300-S10-T10-2	Textile rupture	492.463	1.323
L300-S10-T10-3	Textile rupture	510.827	1.242
L100-S20-T10-1	Pulling out	308.495	1.135
L100-S20-T10-2	Compound mode	341.607	1.077
L100-S20-T10-3	Pulling out	328.276	0.932
L200-S20-T10-1	Compound mode	453.142	0.912
L200-S20-T10-2	Pulling out	427.609	1.065
L200-S20-T10-3	Pulling out	421.903	1.017
L300-S20-T10-1	Textile rupture	471.582	0.953
L300-S20-T10-2	Textile rupture	466.365	0.784
L300-S20-T10-3	Textile rupture	497.148	0.708
L100-S10-T16-1	Compound mode	366.852	1.037
L100-S10-T16-2	Compound mode	396.473	1.178
L100-S10-T16-3	Pulling out	375.391	1.223
L200-S10-T16-1	Compound mode	496.502	1.065
L200-S10-T16-2	Compound mode	477.346	1.109
L200-S10-T16-3	Compound mode	479.157	1.093
L300-S10-T16-1	Textile rupture	533.864	0.769
L300-S10-T16-2	Textile rupture	492.451	0.966
L300-S10-T16-3	Textile rupture	523.704	0.806
L100-S20-T16-1	Pulling out	341.742	1.137
L100-S20-T16-2	Compound mode	370.813	1.026
L100-S20-T16-3	Pulling out	361.031	1.119
L200-S20-T16-1	Compound mode	468.537	0.936
L200-S20-T16-2	Compound mode	491.365	1.132
L200-S20-T16-3	Compound mode	461.033	1.157
L300-S20-T16-1	Textile rupture	508.469	0.824
L300-S20-T16-2	Textile rupture	482.764	0.963
L300-S20-T16-3	Textile rupture	523.113	0.784

**Table 3:** Characteristic values in bond-slip model.

ID	$\tau_{max}$	$S_{max}$	$\tau_{p}$	$S_{p}$
L100-S10-T10	0.302	1.028	0.058	3.813
L200-S10-T10	0.188	1.056	0.054	3.526
L300-S10-T10	0.165	1.251	-	-
L100-S20-T10	0.181	1.048	0.054	3.249
L200-S20-T10	0.120	0.998	0.023	2.014
L300-S20-T10	0.113	0.815	-	-
L100-S10-T16	0.316	1.146	0.073	3.163
L200-S10-T16	0.208	1.089	0.061	1.475
L300-S10-T16	0.169	0.842	-	-
L100-S20-T16	0.198	1.094	0.038	2.577
L200-S20-T16	0.131	1.075	0.018	3.716
L300-S20-T16	0.125	0.857	-	-

## Data Availability

Data sharing not applicable.
